# Socio-economic factors associated with maternal health-seeking behaviours among women from poor households in rural Egypt

**DOI:** 10.1186/s12939-014-0111-5

**Published:** 2014-11-25

**Authors:** Lenka Benova, Oona MR Campbell, Hania Sholkamy, George B Ploubidis

**Affiliations:** Faculty of Epidemiology and Population Health, London School of Hygiene and Tropical Medicine, Keppel Street, London, WC1E 7HT UK; Social Research Center, American University in Cairo, Cairo, Egypt; Centre for Longitudinal Studies, Institute of Education, London, WC1H 0AL UK

**Keywords:** Maternal health, Egypt, Ante-natal care, Facility delivery, Health-seeking behaviour, Poverty

## Abstract

**Introduction:**

Socio-economic inequalities in basic maternal health interventions exist in Egypt, yet little is known about health-seeking of poor households. This paper assesses levels of maternal health-seeking behaviours in women living in poor households in rural Upper Egypt, and compares these to national averages. Secondly, we construct innovative measures of socio-economic resourcefulness among the rural poor in order to examine the association between the resulting variables and the four dimensions of maternal health-seeking behaviour.

**Methods:**

We analysed a cross-sectional survey conducted in Assiut and Sohag governorates in 2010–2011 of 2,242 women in households below the poverty line in 65 poorest villages in Egypt. The associations between four latent socio-economic constructs (socio-cultural resourcefulness, economic resourcefulness, dwelling quality and woman’s status) and receipt of any antenatal care (ANC), regular ANC (four or more visits), facility delivery and private sector delivery for women’s most recent pregnancy in five years preceding survey were assessed using multivariate logistic regression.

**Results:**

In the sample, 58.5% of women reported using any ANC and 51.1% facility delivery, lower than national coverage (74.2% and 72.4%, respectively). The proportion of ANC users receiving regular ANC was lower (67%) than nationally (91%). Among women delivering in facilities, 18% of women in the poor Upper Egypt sample used private providers (63% nationally). In multivariate analysis, higher economic resourcefulness was associated with higher odds of receiving ANC but with lower odds of facility delivery. Socio-cultural resourcefulness was positively associated with receiving any ANC, regular ANC and facility delivery, whereas it was not associated with private delivery care. Dwelling quality was positively associated with private delivery facility use. Woman’s status was not independently associated with any of the four behaviours.

**Conclusions:**

Coverage of basic maternal health interventions and utilisation of private providers are lower among rural poor women in Upper Egypt than nationally. Variables capturing socio-cultural resourcefulness and economic resourcefulness were useful predictors of ANC and facility delivery. Further understanding of issues surrounding availability, affordability and quality of maternal health services among the poor is crucial to eliminating inequalities in maternal health coverage in Egypt.

## Introduction

Egypt witnessed large improvements in maternal health outcomes in recent decades. Maternal mortality ratio decreased from 230 to 66 per 100,000 live births between 1990 and 2010 [1], partly as a consequence of the steadily increasing coverage of preventive and curative interventions. Between 1992 and 2008, the proportion of births covered by regular antenatal care (four or more ANC visits during the pregnancy) increased from 23% to 66%. Nationally, the percentage of births that took place in a health facility increased from 27% in 1992 to 72% in 2008 [2]. However, a complex dynamic underlies these overall trends. Perceived poor quality and inconsistent services provided by the public sector has led to increasing use of private providers. This has resulted in an increase in out-of-pocket health expenditures, which can have a devastating impact on the precarious economic situation of the large proportion of households living near or below the poverty line [3].

Living standards or social hierarchy, captured through various measures of socio-economic position (SEP), are associated with health outcomes in virtually every context where they have been studied [4]. This association has been hypothesised to arise based on the five components of the direct pathway (utilisation of healthcare, psychosocial stress, environmental hazards, health knowledge and lifestyle behaviours) [5-8]. Health-seeking behaviour, representing decisions and actions to seek help from the healthcare system, one of the direct causes, is most amenable to relatively rapid change through policy interventions. An analysis of the sequence of decisions and underlying determinants of choices, from approaching a care provider to the expenditure incurred, is crucial to understanding decision-making mechanisms within households, many of which necessitate stark trade-offs between healthcare-related and other essential household expenditures.

A systematic review of literature published in the previous two decades assessed SEP gradients in maternal health-seeking behaviours in Egypt [9]. Among the six included studies, three dimensions of ANC health-seeking (whether care was received, provider type and intensity of care) and one dimension of delivery health-seeking (provider type) were examined. There is a limited understanding of individual and household-level determinants which may be associated with progression through the various dimensions of health-seeking in poor households. The proportion of Egyptians living below the poverty line increased from 16.7% to 26.3% between 2000 and 2012/2013, with most of the increase seen in rural areas [10]. Upper Egypt is home to 25% of the country’s population, but accounts for 66% of the extremely poor. We hypothesise that ‘traditional’ measures of SEP will have limited utility in differentiating socio-economic environments of poor households and therefore as determinants of health-seeking behaviour.

The current paper contributes to the research on health inequalities in Egypt by analysing the determinants of health-seeking behaviour among poor households in Upper Egypt. The objective of this paper is three-fold. Firstly, we compare the national levels of health-seeking behaviour for maternal care with those reported by women living in households below the poverty line in rural Upper Egypt. Secondly, we construct innovative measures of socio-economic position among the rural poor by broadening the understanding to encompass resourcefulness. Thirdly, we examine the association between the resulting variables capturing SEP and maternal health-seeking behaviour. We hypothesise that the higher the socio-economic resourcefulness, the higher the odds of receiving maternal care. We also hypothesise that utilization of private care will be positively associated with a construct capturing economic or financial resourcefulness. The main contribution of this study stems from conceptualising dimensions of SEP beyond traditional indicators such as asset ownership and educational achievement. Based on Hausmann-Muela’s suggestion that “[t]o a great extent, health-seeking of households depends on their capacity and possibility at a specific moment to mobilise resources, both in material and social or symbolic terms”[11], we aim to capture the operationalization of scarce resources available to poor households. Such detailed analysis of determinants of seeking maternal care is crucial in order to design targeted interventions to address the remaining gap in the coverage of these essential health services.

## Methods

### Data source: households living below poverty line in rural Upper Egypt

A cross-sectional survey, conducted between November 2010 and January 2011 in 65 of the poorest villages in two poorest governorates in Egypt (Assiut and Sohag), was based on a stratified random sample selected from among 25,200 families who applied to and fulfilled the eligibility criteria for the conditional cash transfer program (children of school-age in households). The Ministry of Social Solidarity proxy means testing formula, which contains 17 components (household size and composition, dwelling characteristics, asset ownership, education and occupation status, consumption of utilities and geographic location) was used to include only households living below the poverty line. The sample in our analysis consists of 2,242 women who reported giving birth in the five years preceding the survey and who were not pregnant at the time of the survey. Comparisons with health-seeking behaviours among various groups of women captured on the 2008 Egypt Demographic and Health Survey (DHS) were conducted. The DHS is used to show health-seeking behaviours for three samples: all women (nationally representative sample), women living in Upper Egypt governorates, and women in the poorest DHS wealth quintile living in Upper Egypt.

### Ethical approval

The analysis of these data was approved by the Research Ethics Committee of the London School of Hygiene and Tropical Medicine, UK. Collection of the Upper Egypt dataset was approved by the Institutional Review Board of the American University in Cairo.

### Exposure: measures of socio-economic position

In order to identify variables which might capture socio-economic position, we compiled a list of traditional variables used in our previous analysis of the nationally-representative Demographic and Health Survey (DHS, 2008) and identified in the systematic literature review [9]. Additionally, we conducted a broad literature review in order to identify potential variables capturing household-level utilisation of resources and which were available in the Upper Egypt dataset. This wide literature review spanned gender and women’s studies with particular focus on Middle East contexts [12-15] and development [16,17]; economic literature examining household dynamics [18] and intra-household allocation of resources [19-21] as well as global experience from evaluations of social policy and protection programmes [22,23]. This approach led us to identify 63 variables available in the dataset.

### Outcome: maternal health-seeking behaviours

**Figure 1:**
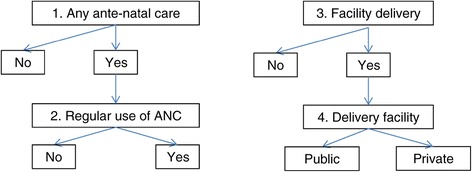
**Four dimensions of health-seeking behaviours analysed. **All women with birth in recall period: Health-seeking behaviour of most recent birth.

Ante-natal care: Utilisation of ANC for the most recent pregnancy were assessed in two ways (Figure 1). The first was a binary variable indicating whether the woman received any ANC during the pregnancy. If ANC was utilised, a second binary variable described whether regular ANC care, consisting of four or more visits during pregnancy was received or not.

Delivery care: We used two health-seeking behaviour outcomes to describe women’s utilisation of delivery care for their most recent delivery (Figure 1). Binary variables captured firstly, whether or not the most recent delivery in the 5-year recall period occurred in a health facility or not; and secondly, among the subset of women with facility deliveries, whether or not private provider was used for this care. The definition of private providers included all non-public sector providers (private hospitals/clinics, private doctor’s offices and other private medical facilities, including NGOs).

### Confounders

A priori confounders of the association between SEP and maternal health-seeking behaviours were identified from published literature [24,25]. These included woman’s age group (in 5-year age intervals) and the number of children (<18 years old) residing in the household. In addition, elements of access, and availability of health services were captured by the relative size of villages (small: less than 6,500 inhabitants, medium: 6,500 to 14,499 and large: >14,500) and of the district (*markez*) (small: <249,000; medium: 250,000 to 349,000 and large: >350,000 inhabitants) according to the 2006 population census figures (CAPMAS). ANC utilisation was considered a potential confounder in analyses of delivery care, capturing pre-delivery exposure to pregnancy-related health services and information.

### Statistical analysis

Exploratory and confirmatory factor analysis (EFA, CFA) was conducted to investigate the measurement structure and determine the composition of the latent SEP variables. This approach acknowledges that whereas female heads of household may be primarily responsible for their own health-seeking [26], their decisions are made within - and shaped by - their immediate social environment [12,27]. It is an attempt to capture results of previous intra-household negotiations about the distribution of scarce resources. The variables suggested to reflect the construct of resource management are not new in their use in economics or public health [28], but their inclusion in a latent model with other SEP measures is innovative.

To quantify unobservable constructs, latent variable modelling utilises common variance among observed indicators. This technique disregards variance that is not common from the latent summary, including random error. The aim is to reduce the dimensionality of the observed data, but to retain a good representation within the latent variable identified [29,30]. Latent variables were constructed in Mplus 7.11 using the Weighted Least Squares, Mean and Variance adjusted (WLSMV) estimator. The latent variables were measured in the sample of women who had a birth in the 5-year period prior to the survey. Hence, unlike the DHS wealth index, which assesses wealth in a nationally-representative sample of households, our SEP variables capture the distribution of the constructs in this specific group of women. Factor loadings of each observed variable represent the association between this indicator and the underlying construct. Model fit was assessed with the Comparative Fit Index (CFI), the Tucker Lewis Index (TLI) and the Root Mean Square Error of Approximation (RMSEA). Full information maximum likelihood (FIML) method was used to deal with missing data in the construction of latent variables and all observations with at least one non-missing value in the observed variables were used. Multivariate logistic regression was used to assess the association between the resulting continuous latent variables identified and maternal health-seeking behaviours, adjusted for confounders. We accounted for the complex survey sampling (clustering within villages and sampling weights) by using the *svyset* command in Stata/SEv.13. The proportion of missing data in the outcome variables was minimal and we utilised complete case analysis.

## Results

### Levels of maternal health-seeking behaviour

**Figure 2:**
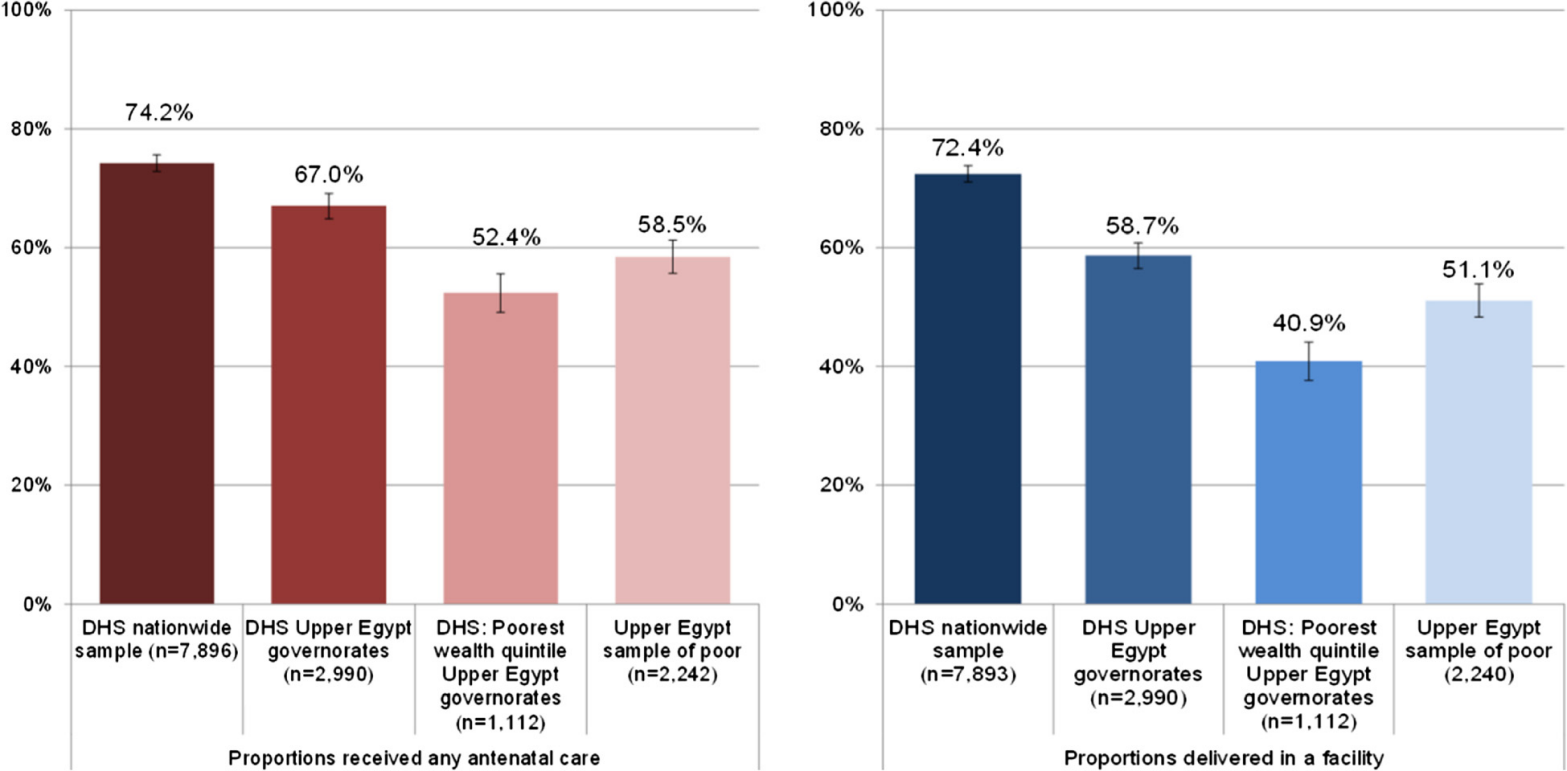
**Levels of ANC utilisation and facility delivery among women with birth in the five-year period preceding the surveys, DHS 2008 and Upper Egypt 2010/11. **DHS - 2008 Demographic and Survey. Complex survey design was accounted for in calculations of proportions and confidence intervals.

**Figure 3:**
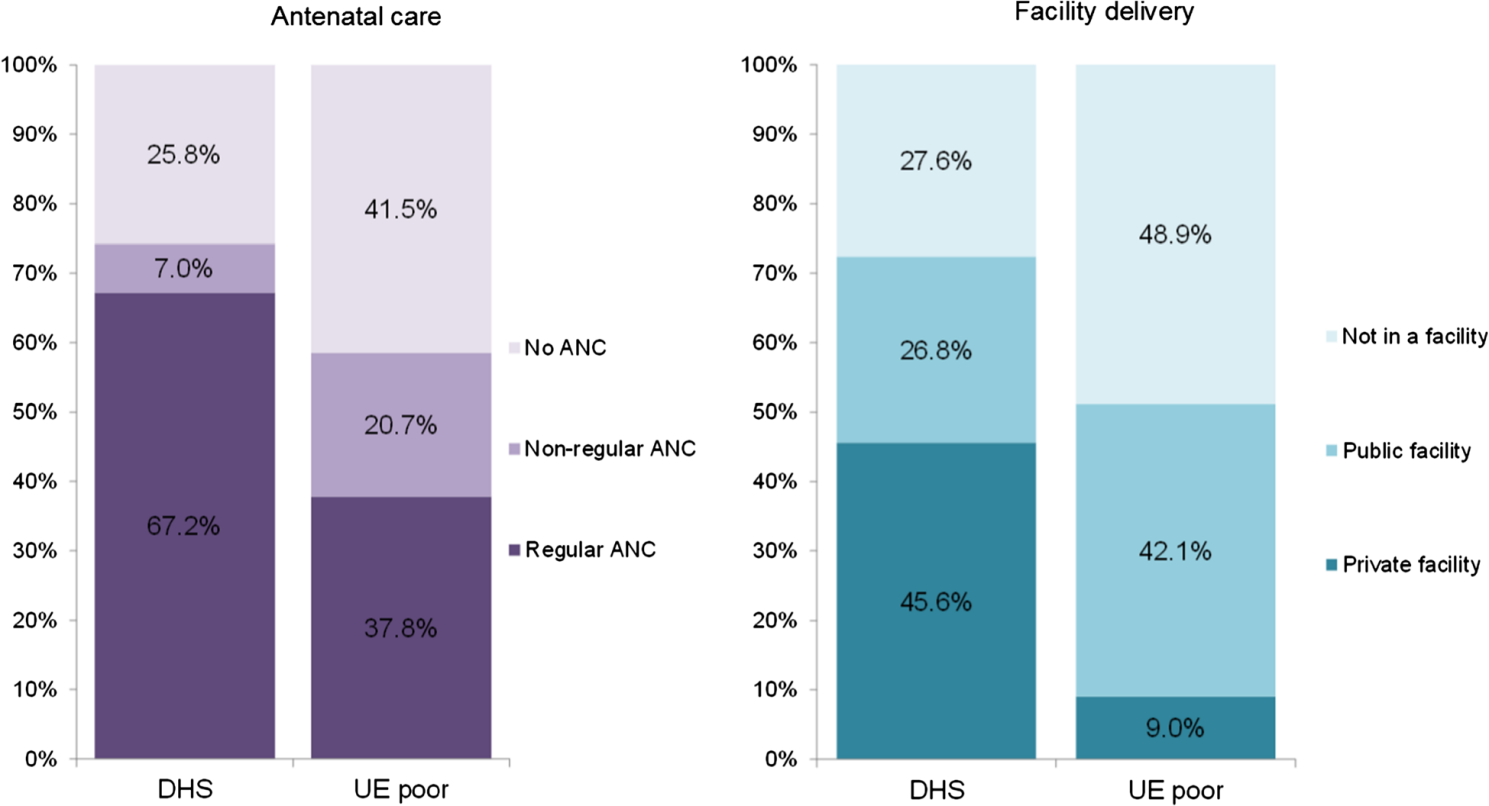
**Proportion of women with a birth in the five-year period preceding the surveys (DHS 2008 and 2010/2011 Upper Egypt [UE]) by maternal health-seeking behaviours. **Regular ANC: 4 or more ANC visits during pregnancy. DHS - Nationally representative sample of women form the 2008 Demographic and Health Survey. Complex survey design was accounted for in calculations of proportions.

Table 1 displays the demographic and socio-economic characteristics of the three analysis groups of women from the Upper Egypt sample of households living below the poverty line. We compared the utilisation of maternal health services among the women from poor households in Upper Egypt to the representative sample in the 2008 DHS. Figure 2 and Table 2 show the proportions of women accessing any ANC and facility-based delivery in four samples: nationally representative DHS sample, DHS sub-sample of women living in Upper Egypt, DHS sub-sample of women in the lowest DHS wealth quintile living in Upper Egypt, as well as the Upper Egypt sample of women from poor households in Assiut and Sohag. The utilisation of both maternal services among the Upper Egypt poor was lower than national levels, but significantly higher than the poorest DHS quintile in Upper Egypt. Figure 3 summarises the four maternal health-seeking behaviours under examination in the sample of the Upper Egypt poor and for comparison, in the nationally representative sample captured by the DHS. Among women with a birth in the five years preceding the surveys, nationally 74.2% (95% CI: 72.8-75.6) reported having received any ANC for their most recent birth and 58.5% (95% CI: 55.6-61.4) in the Upper Egypt sample. Among ANC users, the proportion of women receiving regular ANC was substantially higher in the national DHS sample (91%) compared to 67% in the Upper Egypt sample of poor. In the national sample, 72.4% (95% CI: 70.8-73.9) of women with a birth in the recall period reported delivering in a health facility, 63% of them having used a private provider. In the Upper Egypt sample, 51.1% (95% CI: 46.1-56.0) of women reported delivering in a health facility, but only 18% of these facility deliveries took place in the private sector.

**Table 1:** **Distribution of demographic and socio-economic variables in sample of women with a birth in the five-year recall period (Upper Egypt households living below poverty line)**

**Characteristic**	**Sample of women**	**Women with live birth**	**ANC users**	**Facility delivery users**
	*Weighted sample size*	*2,242*	*1,266*	*1,143*
Woman age group	18-24 (column %)	7.0	8.6	8.3
	25-29	24.3	24.1	26.8
	30-34	26.5	27.9	24.9
	35-39	26.0	26.0	23.4
	40 and above	16.2	13.4	16.6
Number of children	1-2 (column %)	10.5	12.8	13.7
in household	3-4	39.5	38.6	41.9
	5-6	39.5	39.4	35.1
	7 or more	10.5	9.2	9.3
Village size	Small (<6,500) (column %)	18.4	20.3	20.4
	Medium (6,500-14,499)	31.3	29.5	27.8
	Large (>14,500)	50.3	50.2	51.8
District size	Small (<249 K) (column %)	37.2	35.8	32.2
	Medium (250 K-349 K)	26.8	26.1	25.2
	Large (>350 K)	36.0	38.1	42.6
Governorate	Assiut (column %)	60.9	63.3	64.6
	Sohag	39.1	36.7	35.4
Socio-cultural resourcefulness	Mean	−0.002	0.075	0.076
	SE	0.042	0.052	0.052
Economic resourcefulness	Mean	0.057	0.082	−0.012
	SE	0.032	0.036	0.034
Dwelling quality	Mean	−0.020	0.007	0.012
	SE	0.034	0.035	0.037
Woman’s status	Mean	0.015	−0.018	0.031
	SE	0.032	0.035	0.038

SE: standard error. Complex survey design was accounted for in calculations of proportions and sample sizes reported.

K – thousand.

**Table 2:** **Maternal health-seeking behaviours for most recent birth in recall period**

**Health-seeking behaviour outcome** *Ante-natal care (ANC)*		**Variable type**	**Samples and missing data**				**Distribution of outcome in analysed sample and 95% CI**
			**Eligible sample and recall period**	**Eligible sample (size)**	**Missing data (%)**	**Analysed sample (size)**	
**1.**	**Used ANC**	Binary	All women with birth 5 years prior to survey	2,242	-	2,242	58.5% (55.6 - 61.4)
**2.**	**Regular use of ANC (4+ visits)**	Binary	All women with birth 5 years prior to survey who used any ANC	1,312	3.5	1,266	66.9% (63.6 – 70.1)
*Delivery care*							
**3.**	**Delivered in a health facility**	Binary	All women with birth 5 years prior to survey	2,242	<0.01	2,240	51.1% (46.1 – 56.0)
**4.**	**Used private delivery facility**	Binary	All women with birth 5 years prior to survey who delivered in a health facility	1,143	-	1,143	17.7% (13.1 – 23.6)

Complex survey design was accounted for in calculations of proportions and confidence intervals. 95%CI: 95% confidence interval.

### Construction of latent variables capturing socio-economic position

Exploratory factor analysis identified four latent constructs. In confirmatory factor analysis, the latent measurement models for all four constructs had an acceptable fit to the data; the RMSEA level was ≤0.05 and the CFI/TFI ≥0.963, as shown in Table 3. The standardized loading for each observed variable and its standard error are shown in Table 4 (components A,B,C and D).

**Table 3:** **Goodness of fit measures for four latent variables**

**Latent variable**	**CFI**	**TLI**	**RMSEA**
Socio-cultural resourcefulness	0.986	0.971	0.024
Economic resourcefulness	0.974	0.963	0.020
Dwelling quality	0.990	0.970	0.023
Woman’s status	0.995	0.992	0.032

CFI: Comparative Fit Index. TLI: Tucker Lewis Index. RMSEA: Root Mean Square Error of Approximation.

**Table 4:** **Descriptive characteristics of component variables in latent variables, among sample of women who gave birth in 5 year preceding survey (n = 2,254)**

**Component variables**	**Distribution**	**Standardised factor loading (SE)**
**A. Socio-cultural resourcefulness**		
**Woman: years of education**		
Mean (95% CI)	2.50 (2.15-2.84)	0.706 (0.035)
**Woman: literacy**		
Illiterate (column %)	70.7	0.745 (0.034)
Reads/writes with difficulty	8.1	
Reads/writes easily	20.9	
Missing	0.3	
Male head of household: years of education*		
Mean (95% CI)	4.92 (4.55-5.29)	0.670 (0.043)
Male head of household: occupational category*		
Not in employment/no male head of household (column %)	10.9	0.253 (0.032)
Manual or Agricultural worker	73.5	
Higher (Sales, Clerical, Professional)	15.6	
**School enrolment of children 7–18 years old (binary)**		
All children enrolled/No children 7–18 (%)	89.5	0.459 (0.054)
**Identity documents among members of household (binary)**		
All members have identity documents (%)	68.8	0.208 (0.043)
**Percentage of adult household members (18 years+) that ever attended education**		
Mean (95% CI)	49.8 (46.8-52.8)	0.902 (0.037)
**B. Economic resourcefulness (rural)**		
**Household asset ownership (binary)**		
Refrigerator (%)	40.2	0.368 (0.046)
Dwelling is a house or apartment (%)	54.6	0.402 (0.036)
Colour TV (%)	42.7	0.223 (0.034)
Washing machine (%)	67.2	0.238 (0.063)
**Access to means of agricultural production (binary)**		
Own, co-own or rent land (%)	13.1	0.780 (0.036)
Own buffalo (%)	10.2	0.670 (0.046)
Own cows (%)	3.8	0.650 (0.060)
Own horse (%)	13.7	0.735 (0.040)
Own goat or sheep (%)	12.3	0.641 (0.044)
Own poultry (%)	50.2	0.521 (0.030)
**Household consumption (binary)**		
Have access to ration card (%)	56.5	0.405 (0.049)
Youngest child of school age consumed fruit in last 24 hours (%)	15.3	0.236 (0.049)
**C. Dwelling quality**		
**Dwelling utilities (binary)**		
Piped water connection (%)	74.9	0.422 (0.066)
Landline phone (%)	5.9	0.197 (0.063)
Electricity from public network (%)	89.5	0.690 (0.096)
**Construction materials (binary)**		
Floor from cement, tile or plastic (%)	36.8	0.651 (0.063)
Ceiling from concrete or tile (%)	41.6	0.711 (0.073)
Walls from red brick (%)	74.3	0.716 (0.084)
**D. Mobility and decision-making of female head of household**		
**Mobility: leave the house**		
Not allowed (column %)	2.3	0.874 (0.009)
Allowed with permission	70.2	
Allowed with notice/without permission	27.5	
Missing	-	
**Mobility: go to the market**		
Not allowed (column %)	28.6	0.697 (0.021)
Allowed with permission	44.3	
Allowed with notice/without permission	27.1	
Missing	-	
**Mobility: go to hospital**		
Not allowed (column %)	1.4	0.865 (0.011)
Allowed with permission	69.2	
Allowed with notice/without permission	29.4	
Missing	-	
**Mobility: visit relatives**		
Not allowed (column %)	3.5	0.814 (0.013)
Allowed with permission	67.0	
Allowed with notice/without permission	29.5	
Missing	-	
**Mobility: visit neighbours**		
Not allowed (column %)	10.8	0.732 (0.018)
Allowed with permission	46.4	
Allowed with notice/without permission	42.8	
Missing	-	
**Mobility: run an errand**		
Not allowed (column %)	19.6	0.538 (0.028)
Allowed with permission	51.7	
Allowed with notice/without permission	28.7	
Missing	-	
**Decision-making about household budget**		
Someone else (column %)	1.6	0.429 (0.029)
Male head of household solely	51.9	
Female head of household or jointly	46.3	
Missing	0.2	
**Decision-making about visiting friends or family**		
Someone else (column %)	0.6	0.457 (0.038)
Male head of household solely	61.3	
Female head of household or jointly	37.8	
Missing	0.3	

*87 households have no male head of household – values coded as missing.

No missing data.

95% CI: 95% confidence interval SE: Standard error svyset applied in calculation of distribution. No missing data.

Firstly, we identified a latent measure of *socio-cultural resourcefulness*, which was constructed of seven observed variables. This included four traditional variables (woman’s years of education, woman’s literacy level, male head of household years of education, male head of household occupational category) and three innovative indicators (school enrolment of school-age children, possession of identity documents by members of the household and proportion of all adult member of the household who had ever attended school). The mean standardised socio-cultural resourcefulness score was −0.002 (standard error [SE]: 0.042, range: −1.383 to 2.039); the higher the factor score, the higher the socio-cultural resourcefulness. The observation with a median socio-cultural resourcefulness score was characterised as an illiterate woman with no education, with a husband in manual or agricultural occupation with five years of education, in a household where all children of school age are enrolled in school, all household members possess identity documents and where 50% of adult members had attended school at some point.

Secondly, measurement of rural *economic resourcefulness* was based on twelve variables, ownership of four household assets (fridge, colour TV, washing machine and house/apartment), agricultural assets (access to land and ownership of five types of domestic animals) as well as two innovative indicators (possession of a ration card and fruit consumption of the youngest child of school age in the 24 hour period preceding the survey). The mean standardised economic resourcefulness score was 0.057 (SE: 0.032, range: −1.276 to 2.532) and the higher the factor score, the higher the economic resourcefulness. A median economic resourcefulness score described a household which does not own a fridge or a colour TV, but owns a washing machine (automatic or semi-automatic), lives in a house or apartment which is shared with other households, has no access to land (owned, co-owned or rented), does not own any large domestic animals (buffalo, cows, horses, goats or sheep) but owns poultry, has access to a ration card, and where the youngest child consumed a portion of fruit during the recall period.

Thirdly, we identified a latent variable reflecting six observed characteristics of the *dwelling quality*, including the type of water and electricity connection, availability of a landline telephone, as well as construction materials of the floor, ceiling and walls. The mean standardised dwelling quality score was −0.020 (SE: 0.034, range: −1.692 to 1.213) and the higher the factor score, the higher the dwelling quality. A median dwelling quality score described a household which had a piped water connection and electricity from the public network, but no landline telephone connection, where the floor was not from cement, tile or plastic; the ceiling was not from concrete or tile; and where the walls were constructed from red brick.

Fourthly, eight observed variables describing various aspects of woman’s mobility and decision-making ability reflected a construct which we termed *woman’s status*. The mean standardised woman’s status score was 0.015 (SE: 0.032, range: −2.735 to 1.720) and the higher the factor score, the higher the woman’s status. A woman with a median level of woman’s status score reported being able to go to a hospital without permission, being able to leave the house, visit relatives and run errands with permission, but not being allowed to go to the market or visit neighbours, and who solely or jointly with her husband took decisions about whether to visit friends or family, but whose husband was the sole decision-maker about the household budget.

The correlation matrix between the four latent variables is shown in Table 5 (component A). Among the variables, the only significant association was between socio-cultural resourcefulness and dwelling quality. We assessed the crude associations between the four latent variables and the area-level characteristics (village size, district size and governorate), as shown in Table 5, component B. Socio-cultural resourcefulness was associated with district size; the larger the district, the higher the mean socio-cultural resourcefulness score. Economic resourcefulness was associated with all three area-level variables. The mean score was lower in smaller villages, larger in smaller districts, and higher in Sohag than in Assiut. Dwelling quality score was positively associated with larger village and district sizes, but not with governorate. Woman’s status was not associated with any of the geographic variables.

**Table 5:** **Correlation coefficients between socio-economic position variables and p-values and crude associations between area-level and socio-economic position variables**

		**Socio-cultural resourcefulness**	**Economic resourcefulness**	**Dwelling quality**	**Woman’s status**
**A. Correlation coefficients between socio-economic position variables and p-values**					
**Socio-cultural resourcefulness**		1.0000			
**Economic resourcefulness**		−0.0068 (0.749)	1.0000		
**Dwelling quality**		0.1687 (<0.001)	0.0084 (0.691)	1.0000	
**Woman’s status**		−0.0299 (0.157)	0.0411 (0.052)	0.0028 (0.896)	1.0000
**B. Crude associations between area-level and socio-economic position variables**					
		Mean (SE)	Mean (SE)	Mean (SE)	Mean (SE)
**Village size**	Small (<6,500)	−0.016 (0.082)	−0.058 (0.078)	−0.105 (0.062)	0.085 (0.096)
	Medium (6,500-14,499)	−0.048 (0.078)	0.165 (0.047)	−0.070 (0.087)	−0.063 (0.075)
	Large (>14,500)	0.033 (0.084)	0.032 (0.062)	0.041 (0.054)	0.037 (0.048)
	*p-value*	*0.200*	*<0.001*	*<0.001*	*0.017*
**District size**	Small (<249 K)	−0.085 (0.065)	0.130 (0.042)	−0.080 (0.078)	−0.031 (0.046)
	Medium (250 K-349 K)	−0.028 (0.093)	0.122 (0.061)	0.031 (0.048)	0.009 (0.102)
	Large (>350 K)	0.105 (0.095)	−0.067 (0.070)	0.003 (0.077)	0.066 (0.056)
	*p-value*	*<0.001*	*<0.001*	*0.009*	*0.096*
**Governorate**	Assiut	0.018 (0.075)	−0.024 (0.050)	−0.012 (0.060)	0.019 (0.046)
	Sohag	−0.033 (0.063)	0.185 (0.045)	−0.032 (0.054)	0.008 (0.066)
	*p-value*	*0.216*	*<0.001*	*0.564*	*0.791*

SE – standard error. P-value of ANOVA.

### Determinants of maternal health-seeking behaviour in Upper Egypt poor

#### Any ANC use

The adjusted analysis of determinants of ANC use showed that both higher socio-cultural resourcefulness and higher economic resourcefulness were associated with increased odds of having received any ANC (Table 6). A one unit increase in socio-cultural resourcefulness was independently associated with a 16% increase in the odds of ANC (OR = 1.16, 95% CI 1.02-1.32) and a one unit increase in economic resourcefulness was associated with a 27% increase in the odds of ANC (OR = 1.27, 95% CI: 1.08-1.49). Women’s status or dwelling quality scores were not associated with receiving ANC. Women in the oldest age group (40 years and above) had significantly lower odds (OR = 0.64, 95% CI: 0.49-0.85) of receiving ANC compared to women in the reference group (35–39 years).

**Table 6:** **Multivariate analysis of determinants of maternal health-seeking behaviours among women in Upper Egypt households living below the poverty line**

**Variable**	**Any ANC**		**Regular ANC**		**Facility delivery**		**Private delivery care**	
**Sample size**	**2,242**		**1,266**		**2,240**		**1,143**	
	***OR (95% CI)***	***p-value***	***OR (95% CI)***	***p-value***	***OR (95% CI)***	***p-value***	***OR (95% CI)***	***p-value***
Socio-cultural resourcefulness*	1.16 (1.02-1.32)	0.022	1.17 (1.01-1.36)	0.038	1.14 (1.02-1.28)	0.028	1.04 (0.85-1.27)	0.705
Economic resourcefulness*	1.27 (1.08-1.49)	0.004	0.97 (0.78-1.19)	0.747	0.81 (0.69-0.95)	0.009	0.91 (0.66-1.25)	0.538
Dwelling quality*	1.10 (0.96-1.26)	0.159	0.95 (0.80-1.12)	0.512	1.08 (0.93-1.26)	0.291	1.48 (1.07-2.04)	0.019
Woman’s status*	0.92 (0.80-1.06)	0.228	0.97 (0.80-1.16)	0.707	1.07 (0.93-1.23)	0.314	0.98 (0.76-1.26)	0.852
**Woman’s age group**								
18-24	1.48 (0.87-2.40)	0.147	0.70 (0.31-1.59)	0.387	0.87 (0.46-1.62)	0.649	1.39 (0.49-3.95)	0.526
25-29	0.91 (0.69-1.20)	0.500	0.83 (0.49-1.43)	0.507	1.05 (0.82-1.36)	0.676	0.52 (0.25-1.09)	0.081
30-34	1.10 (0.82-1.48)	0.503	0.90 (0.59-1.37)	0.619	0.90 (0.67-1.22)	0.516	1.23 (0.77-1.94)	0.380
35-39	1 (ref)		1 (ref)		1 (ref)		1 (ref)	
40 and above	0.64 (0.49-0.85)	0.002	0.89 (0.55-1.44)	0.639	1.45 (1.10-1.91)	0.009	0.76 (0.44-1.29)	0.300
**Number of children**								
1-2	1.62 (0.93-2.84)	0.089	1.10 (0.55-2.22)	0.784	2.07 (1.36-3.14)	0.001	0.80 (0.32-2.02)	0.628
3-4	0.99 (0.79-1.26)	0.953	0.79 (0.57-1.10)	0.156	1.40 (1.12-1.74)	0.003	1.20 (0.73-1.97)	0.456
5-6	1 (ref)		1 (ref)		1 (ref)		1 (ref)	
7 or more	0.79 (0.56-1.11)	0.167	0.87 (0.54-1.40)	0.553	1.06 (0.74-1.51)	0.751	1.06 (0.63-1.78)	0.836
**Governorate**								
Assiut	1 (ref)		1 (ref)		1 (ref)		1 (ref)	
Sohag	0.78 (0.60-1.01)	0.059	1.08 (0.80-1.46)	0.589	0.78 (0.53-1.15)	0.206	1.91 (0.97-3.74)	0.060
**Received any ANC**								
No					1 (ref)		1 (ref)	
Yes					1.96 (1.57-2.45)	<0.001	1.87 (1.16-3.01)	0.011

*Continuous variables; odds ratio associated with one unit increase in score. p-value of Wald test. OR: Odds ratio. 95% CI: 95% confidence interval.

#### Regular ANC use

Women who reported receiving any ANC care comprised the sample for analysis of regular ANC use determinants. In adjusted analysis, socio-cultural resourcefulness was associated with the odds of receiving regular ANC. A one unit increase in socio-cultural resourcefulness was associated with 17% higher odds of regular use of ANC (OR = 1.17, 95% CI: 1.01-1.36). None of the remaining latent variables, demographic or geographic variables were associated with receiving regular ANC.

#### Facility delivery

Economic resourcefulness was associated with facility use for delivery care. The effect of a one unit increase in economic resourcefulness was associated with 19% lower odds of delivering in a facility (95% CI: 0.69-0.95). A one unit increase in socio-cultural resourcefulness was associated with a 14% increase in the odds of facility delivery (95% CI: 1.02-1.28). Dwelling quality or women’s status were not associated with facility delivery. Women in the oldest age group had 45% higher odds (95% CI: 1.10-1.91) of delivering in a health facility compared to women in the reference group. Women living in households with fewer than five children were more likely than those living in households with 5–6 children to deliver in a health facility. Women who reported receiving ANC had nearly double the odds of delivering in a health facility.

#### Private delivery facility use

Among the sample of women who reported delivering in a facility, neither socio-cultural nor economic resourcefulness were associated with the use of private delivery facility. On the other hand, a one unit increase in dwelling quality score was associated with a 48% increase in the odds of private facility use (95% CI: 1.07-2.04). Women’s status, age group or the number of children in household were not associated with private facility use. Residence in Sohag governorate was associated with a nearly two-fold increase in the odds of private delivery facility use compared to residence in Assiut. Lastly, women who received any ANC had 1.87 higher odds (95% CI: 1.16-3.01) of using private delivery facilities compared to women who did not.

In all four models, the inclusion of variables capturing size of the village and district of residence had no meaningful effects on the estimates of association between the four SEP variables and health-seeking behaviour outcomes, and were therefore no included in the final models.

## Discussion

We found that the level of ANC and facility delivery utilisation in the poor Upper Egypt sample was lower than the national average, but higher than reported by the poorest DHS wealth quintile residing in the Upper Egypt region. The proportion of ANC users receiving regular ANC was lower among the Upper Egypt poor than in the nationally-representative sample. The proportion of women who used private delivery care among women who delivered in facilities was 63% in the national sample compared to less than one fifth among the Upper Egypt poor. In this paper, we conducted exploratory factor analysis of potential indicators and constructed four latent variables: socio-cultural resourcefulness, economic resourcefulness, dwelling quality and woman’s status. We identified socio-cultural resourcefulness as capturing the socio-cultural capital of the woman whose maternal health-seeking behaviour as well as additional characteristics of the household. Economic resourcefulness reflected the unobserved construct of accumulated resources available to rural households [31,32]. Together, they described the individual and household-level factors that are hypothesised to be associated with the process of choice related to pregnancy and delivery care [33].

The character of ANC is largely preventive and conducted on an outpatient/clinic basis, while facility delivery care is inpatient, intensive and potentially invasive. Differences in perceptions of the need for ANC and facility delivery may help explain the fact that whereas increasing economic resourcefulness was associated with higher odds of receiving ANC, it was associated with lower odds of facility delivery. Socio-cultural resourcefulness was positively associated with receiving any ANC, regular ANC and facility delivery, whereas it was not associated with private delivery care. Women with higher dwelling quality scores were more likely to deliver in a private facility, possibly capturing the more urbanised character of locations that are attractive to private health providers and greater availability of such services. Woman’s status was not independently associated with any of the four health-seeking behaviours. The importance of exposure to health services and pregnancy information is exemplified in the strong association between receiving ANC and the odds of facility delivery. However, the content and quality of the interaction between the pregnant woman and the health professional and resulting expectations from health providers needs to be better understood in light of our finding that receipt of any ANC also predicted private delivery care.

### Strengths and limitations

#### Study type and participants

This study was conducted on a sample of poor households residing in Upper Egypt which applied to a conditional cash transfer program and fulfilled its eligibility criteria. Therefore, the levels of maternal health-seeking cannot be interpreted as being representative of all Assiut and Sohag households living below the poverty line, as the sample was unlikely to have captured the most marginalised and vulnerable poor households. However, the households captured in this dataset are potentially the type of households that would be more likely to participate in programmes designed to improve coverage of essential care, for example those related to maternal and child health. Data was collected in a cross-sectional survey conducted in 2010/11 with a high overall response rate of 97.1%. To capture the most up-to-date patterns of health-seeking behaviour, we analysed the circumstances surrounding the most recent birth in the five-year recall period. However, this analysis faced several limitations. The cross-sectional and observational design of this study limited our ability to assess causal relationships between SEP and health-seeking behaviours.

#### Measurement of exposures

The four latent SEP measures constructed and used in this study relied on observed self-reported variables, which are easy to collect and process but may present some risk of reporting bias due to the particular context of data collection. The dataset used contained low proportions of missing data in the component variables of each SEP measure.

#### Measurement of outcomes

All measures of health-seeking behaviour analysed in this study were self-reported. Whereas we expect the report of the occurrence of a live birth in the recall period to be reliable, the health-seeking behaviour variables may be impacted by measurement error and recall bias.

#### Statistical model

The main strength of this study stems from the inclusion of both socio-cultural and economic measures in the model predicting their association with various dimensions of maternal health-seeking behaviour. While we attempted to identify and include all potential confounders, the presence and extent of unmeasured confounding cannot be completely ruled out. There are several potential sources of unmeasured confounding. Data on women’s parity was not collected. Our analysis of the 2008 DHS showed that age group is strongly associated with parity among women living in Upper Egypt [34]. As proxies for parity, we used woman’s age group and number of children living in her household. Potentially, the number of children, in addition to being strongly correlated with parity, also captured the extent of the woman’s ability to be absent for the duration of receiving care. Information on the existence of pregnancy complications, which had previously been shown to be positively associated with maternal care utilisation, was not collected and thus could not be used in the adjusted analysis.

#### Consideration of health services availability and quality

Village size, district size and governorate variables were considered for inclusion in the analysis as potential confounders because they are associated with the extent of urbanisation (components in both economic resourcefulness and dwelling quality). For instance, districts with larger populations might have a higher number and higher level of health facilities compared to smaller districts, potentially reducing the direct and indirect costs of obtaining maternal care. We were unable to access more detailed data on health facilities (location, level, public or private ownership) in order to describe these potential of association more precisely. Moreover, information about the existence of health facilities does not necessary mean that such facilities are functioning and providing health services. Localised information on quality and reliability of health services in Egypt was not available.

## Conclusion

Socio-economically structured inequities in health coverage play a significant role in gaps in maternal health coverage, globally and in Egypt [35-38]. Further improvements in maternal health indicators are therefore highly dependent on increasing coverage among the poorest and most disadvantaged segments of society [39-41]. In this paper, we found that the construction of socio-economic position measures among households living below the poverty line necessitated the use of indicators capturing resourcefulness. The resulting latent variables of socio-cultural and economic resourcefulness were useful predictors of receiving ANC and delivery care, although effect sizes were modest. However, in order to better understand characteristics of maternal care (i.e., regularity of ANC and choice of provider for delivery care), multidisciplinary research is needed to explore issues surrounding availability, accessibility and quality of maternal health services, as well as cultural attitudes.

Analysis of social and economic determinants of health behaviours and health outcomes has progressed from a limited descriptive approach to focusing on identifying determinants which may be amenable to intervention. The latter approach is needed especially in contexts with a substantial proportion of the population living in poverty, such as Egypt, in order to design targeted and effective interventions to improve health status. Programmes designed to narrow the gap in reproductive and maternal care utilisation have been implemented in many countries. These include, for example, voucher schemes and monetary rewards for utilisation of pregnancy and delivery care [42-44], removal of user fees [45,46], creation of community or micro health insurance [47,48], women’s groups [49,50], conditional cash transfers [51-53], birth preparedness planning [54], provider social franchising [55,56], mobile banking and savings schemes [57], and private provider reimbursement [58]. While these interventions may target or affect both the demand for and the supply and quality of maternal health services, their results thus far have been equivocal [59].

In light of the state of research in this field, our analysis opens another avenue for reflection. We attempted to enhance the understanding of the individual and household circumstances which enable women from poor households to seek maternal care. With the use of expanded understanding of individual and household-level SEP in rural areas, we showed that socio-economic circumstances are important predictors of maternal health-seeking behaviour alongside other known factors such as age and geographic location. We also found that women’s status, encompassing mobility and decision-making, appeared to matter less in these processes than socio-cultural and economic resources. This is perhaps because motherhood is a more ‘traditional’ empowerment resource that is governed by different cultural rules and norms than those that indicate development markers of women’s autonomy or empowerment. However, we must approach analysis of health-seeking behaviour as a set of discreet dimensions – for example, by separating the use of any ANC from the use of timely and regular ANC. The interventions that can potentially increase the proportion of women accessing any ANC may be different from those encouraging early and regular ANC among ANC users. In this regard, our analysis of the 2008 DHS showed that other considerations, such as preference for private care, may be driving expenditures on care that among the poor could reach catastrophic proportions [34]. We believe that future research may be able to build on our findings by approaching poor households in a more nuanced perspective.
